# Clinical Disease and Outcomes of Invasive Staphylococcus lugdunensis Infection in a University Hospital in Saudi Arabia

**DOI:** 10.7759/cureus.52103

**Published:** 2024-01-11

**Authors:** Reham Kaki

**Affiliations:** 1 Internal Medicine, Infectious Disease & Infection Control, King Abdulaziz University (KAU) Hospital, Jeddah, SAU

**Keywords:** staphylococcus lugdunensis, risk factors, mortality, infection, comorbidity, antibiotic susceptibility

## Abstract

Background

*Staphylococcus lugdunensis* is a pathogen that can cause various diseases in humans, of which bacteremia and infective endocarditis have been described most extensively. In Saudi Arabia, reports of *S. lugdunensis* infection are extremely rare, and no studies have reported *S. lugdunensis* antibiotic susceptibility. The objective of this study was to determine *S. lugdunensis* clinical disease, potential risk factors, susceptibility pattern, and 30-day mortality.

Methods

A retrospective study was performed at King Abdulaziz University Hospital in Jeddah, Saudi Arabia, from January 1, 2015, to December 31, 2022. Patients ≥14 years old were included. All variables, such as age, sex, body mass index (BMI), clinical manifestations, source of infection, antimicrobial susceptibility, antimicrobial given, duration of treatment, and 30-day mortality, were obtained from electronic health charts.

Results

Twenty-five patients with *S. lugdunensis* infection were identified, with a median age of 58 years and all had comorbidities (mean: 2, range 1-10). The patients had a median BMI of 28, and most patients were either overweight (28%, n = 7) or obese (48%, n = 12). The 30-day mortality was only 8% (n = 2). *S. lugdunensis* was most often cultured from wound swabs (72%, n = 18) and blood (20%, n = 5). The majority (68%, n = 17) of infections were community-acquired. Antibiotic susceptibility to vancomycin was 100% (n = 25), oxacillin 72% (n = 18), and clindamycin and trimethoprim-sulfamethoxazole 64% (n = 16) each. The mean Charlson comorbidity index was significantly higher (p-value = 0.027) among the deceased patients (6.00 ± 2.12) than those that survived (1.83 ± 1.77).

Conclusion

*S. lugdunensis *can cause clinically significant disease, especially in patients with multiple comorbidities, and a higher Charlson comorbidity index was found in patients who died.

## Introduction

*Staphylococcus lugdunensis* was initially thought to be part of the normal skin flora, but it has since been recognized as an important human pathogen. Cases of, among others, native valve endocarditis, prosthetic valve endocarditis, skin and soft tissue infection, primary bacteremia, urinary tract infection (UTI), bone and joint disease, and prosthetic joint infection have been reported [1]. *S. lugdunensis* is a coagulase-negative *Staphylococcus *species but behaves more like the coagulase-positive *Staphylococcus aureus* due to the presence of several virulence factors such as various proteins that facilitate adherence to collagen, laminin, fibrinogen, vitronectin, and fibronectin. Other virulence factors of *S. lugdunensis* are lysozyme resistance, lipase, DNase, and the ability to form biofilms [1-6]. *S. lugdunensis* has also been found to produce von Willebrand factor binding protein which plays a prominent role in causing native valve infective endocarditis [7].

Most *S. lugdunensis* studies focused on bacteremia and infective endocarditis [8-10], while few discussed other diseases caused by this pathogen. The mortality rate of patients with coagulase-negative *Staphylococcus* infections ranges between 8 and 14%. No mortality rates are available for *S. lugdunensis* infections in general, but mortality rates due to *S. lugdunensis* endocarditis were found to be high at 42-70% [1,11-13]. The majority of *S. lugdunensis* clinical diagnoses (55.4%) were skin infections, while a study of skin and post-surgical wound infections due to *S. lugdunensis* found that these infections were found mainly below the waist (73%) [14,15].

*S. lugdunensis* has a different antimicrobial sensitivity profile than other coagulase-negative *Staphylococcus* species as it remains susceptible to most antibiotics, and resistance to penicillin is as low as 15% in Sweden and 25% in Denmark, although reportedly higher (45%) in the United States and in Taiwan (87%) [16-19]. Resistance to clindamycin and erythromycin is also low [18-20].

In Saudi Arabia, reports of *S. lugdunensis* infections are extremely rare [21,22], as it constitutes only a small proportion of coagulase-negative *Staphylococcus* species, and no studies reported *S. lugdunensis* antibiotic susceptibility in Saudi Arabia. This retrospective study aimed to determine *S. lugdunensis* clinical disease, risk factors, susceptibility pattern, and 30-day mortality in Saudi Arabia.

## Materials and methods

Study design and patient selection

This was a retrospective study at King Abdulaziz University Hospital in Jeddah, Saudi Arabia, which has 1000 beds. The study investigated *S. lugdunensis* clinical disease, risk factors, susceptibility pattern, and mortality. We included all patients with *S. lugdunensis*-positive isolates from all types of clinical specimens submitted to the laboratory from January 1, 2015, to December 31, 2022. 

We included patients ≥14 years old as adolescents are transferred from pediatric to adult care at 14 years of age. We also excluded patients with positive urine cultures without clinical disease, as these were likely asymptomatic bacteriuria rather than UTI, and patients with positive swabs without clinical disease, as those were more likely colonized rather than infected. Finally, we also excluded patients with only a single positive blood culture without evidence of clinical disease, such as fever (≥38°C), hypotension (defined as systolic blood pressure <90 mm Hg), or leukocytosis (white blood cell count >11K/μL) as this might represent blood contamination at the time of collection rather than true bacteremia.

Data collection

All *S. lugdunensis* isolates were identified using Gram staining and biochemical methods, i.e., catalase-positive, coagulase-negative, ornithine decarboxylase-positive, and pyrrolidonyl arylamidase-positive. The identification of the isolates was confirmed with matrix-assisted laser desorption ionization/time of flight mass spectrometry and the Biotyper 2.0 database (Bruker Daltonics, Bruker, Massachusetts, U.S.), as well as with analysis of the presence of the *tanA* gene by polymerase chain reaction. Confirmed *S. lugdunensis* isolates subsequently underwent antimicrobial susceptibility testing according to the guidelines of the Clinical & Laboratory Standards Institute. This was performed with the automated bacterial identification and susceptibility testing system VITEK-2 (bioMérieux, France) using card AST-ST03.

We reviewed the electronic health charts of the patients for age, sex, body mass index (BMI), clinical manifestations, source of infection, antimicrobial susceptibility, antimicrobial given, duration of treatment, and 30-day mortality. All data were entered into an electronic database. Healthcare-associated infection was defined as an infection that was acquired after hospitalization as it developed at ≥48 hours after admission. All infections that were present at the time of admission or developed ≤48 hours of admission were termed community-acquired.

Statistical analysis

Categorical variables were presented as frequencies and percentages. Central tendencies for the numerical variables were presented. The numerical variables mainly were non-normally distributed. The distributions were checked by the Kolmogorov-Smirnov test, histogram, and skewness values. The correlation between numerical variables was assessed by the Spearman Rank Correlation test. Chi-square tests assessed the association between gender, acquisition, type of infection, and mortality. Mann-Whitney U tests assessed the relationship between age, Charlson comorbidity, duration of antibiotic treatment, and mortality. All analyses were performed with IBM SPSS Statistics for Windows, Version 24 (Released 2016; IBM Corp., Armonk, New York, United States). A p-value <0.05 was considered statistically significant.

## Results

Patients

**Figure 1:**
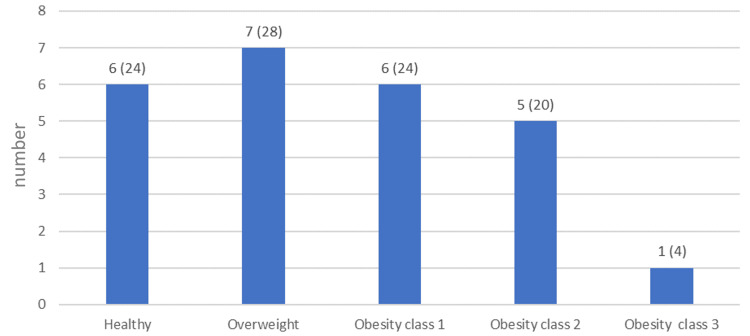
BMI categories in the patients Number (and percentage) of patients within each BMI category. A healthy BMI is defined as a BMI ≥18.5 to <25, and overweight is defined as BMI ≥25 to <30. Obesity categories are defined as Class 1: BMI ≥30 to < 35, Class 2: BMI ≥35 to < 40, and Class 3: BMI ≥40. BMI, body mass index.

This retrospective chart review study included 25 cases of *S. lugdunensis* infection. A little over half (56%) of the patients were females (Table 1). The median age of the patients was 58 years, and their median BMI was 28 kg/m^2^. Presenting the patients' BMIs in BMI categories showed that most of the patients were either overweight (28%) or obese (48%) (Figure 1). Two of the patients died within 30 days of diagnosis (8%).

**Table 1:** Demographics and clinical data AST, aspartate aminotransferase; BMI, body mass index; INR, international normalized ratio; N, number

Numerical variables		Mean	Median	Standard deviation	Reference range
Age, in years		55.12	58.00	17.11	
BMI, in kg/m^2^		30.51	28.00	8.03	
Charlson weighted comorbidity index		2.16	2.00	2.13	
White blood cell count, in 10^3^/μL		10.53	9.00	5.34	4.5 – 11.5
Platelet count, in 10^3^/μL		312.46	313.50	138.81	150 – 450
AST, in U/L		28.09	23.00	20.31	15 – 37
Bilirubin, in μmol/L		9.87	8.00	6.86	0 – 17
INR		1.10	1.00	0.17	
Creatinine, in µmol/L		124.56	70.00	151.72	53 – 115
Duration of antibiotic treatment, in days		11.25	7.50	9.06	
Categorical variables	Attributes		N	%	
Gender	Female		14	56.0	
	Male		11	44.0	
Acquisition	Community		17	68.0	
	Hospital		8	32.0	
Type of infection	Monomicrobial		16	64.0	
	Polymicrobial		9	36.0	

Infection acquisition and strain culture

The most common site where *S. lugdunensis* was cultured from was wound tissue in 18 patients (72%), followed by blood culture in five patients (20%). In the other patients,* S. lugdunensis *was cultured from urine (n = 1, 4%) and synovial fluid (n = 1, 4%). In the 17 patients with skin and soft tissue infections, all of these infections were below the lower abdomen, in the perineal area, and on the lower limbs. Most patients had community-acquired (n = 17, 68%) rather than hospital-acquired infections, and monomicrobial (n = 16, 64%) rather than polymicrobial cultures (Table 1).

Comorbidities

**Figure 2:**
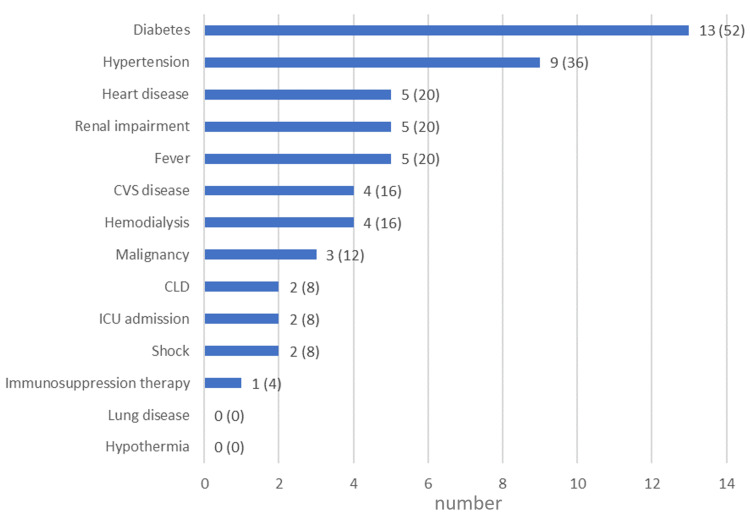
Comorbidities, signs, symptoms, and immunosuppression in the patients Number (and percentage) of patients with comorbidities, signs, symptoms, and immunosuppression. Of the three patients with malignancies, two had hematological malignancies (diffuse B cell lymphoma and T cell lymphoma) one of which was treated with chemotherapy, and one had a solid organ tumor (pancreatic cancer). CLD, chronic liver disease; CVS, cardiovascular disease; ICU, intensive care unit.

All patients had comorbidities (mean: 2, range: 1-10). The most common comorbidity was diabetes (n = 13, 52%), followed by arterial hypertension (n = 9, 36%). None of the patients had lung disease or hypothermia (Figure 2). Their median Charlson comorbidity index score was 2 (Table 1).

Antibiotic susceptibility and prescription

**Figure 3:**
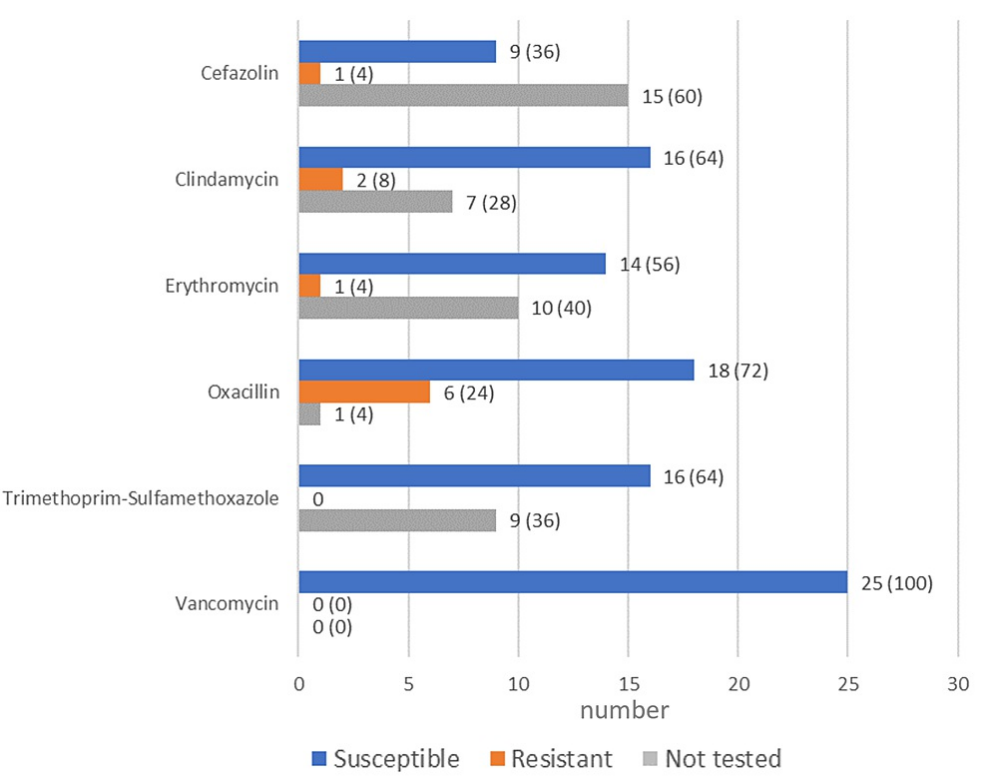
Susceptibility to antibiotics of the S. lugdunensis strains Number (and percentage) of the *S. lugdunensis *strains susceptible, resistant, or not tested for each antibiotic. Antibiotics are in alphabetical order.

**Figure 4:**
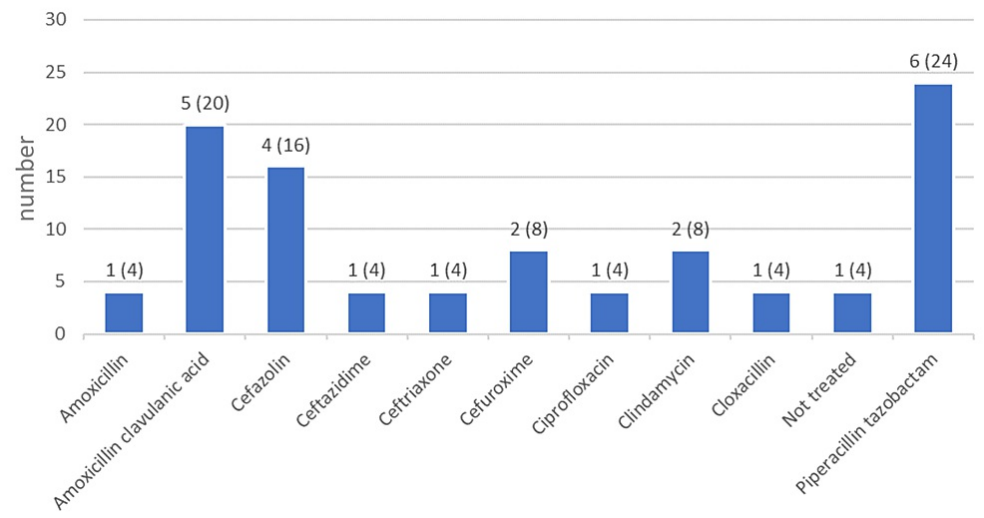
Antibiotics prescribed to the patients Number (and percentage) of patients treated with each antibiotic.

The S*. lugdunensis* strains were tested for antibiotic susceptibility. The highest percentages of antibiotic susceptibility were seen for vancomycin (n = 25, 100%) and oxacillin (n = 18, 72%), followed by clindamycin and trimethoprim-sulfamethoxazole at 64% (n = 16) each. The highest resistance was found for oxacillin at 24% (n = 6) (Figure 3). Piperacillin tazobactam was the most commonly prescribed antibiotic (n = 6, 24%), followed by the combination of amoxicillin and clavulanic acid (n = 5, 20%) (Figure 4). The median duration of antibiotic treatment was 7.5 days (Table 1).

Associations with mortality

There were no statistically significant associations between gender, infection acquired, type of infection, and mortality, as all p-values were > 0.050 (Table 2). The Charlson comorbidity index was significantly higher among the deceased group (6.00 ± 2.12) versus the alive group (1.83 ± 1.77), p-value = 0.027. Age and duration of antibiotic therapy were not significantly different between these two groups, with p-values of 0.200 and 0.587, respectively (Table 3).

**Table 2:** Association between mortality and gender, acquisition, or type of infection *based on Chi-square tests. N, number

Variables	Mortality, n/N (%)	p-value*	Odds ratio
Gender		0.859	0.769
Female	1/14 (7.1%)		
Male	1/11 (9.1%)		
Acquisition		0.569	0.438
Community-acquired	1/17 (5.9%)		
Healthcare-associated	1/8 (12.5%)		
Type of infection		0.667	0.533
Monomicrobial	1/16 (6.3%)		
Polymicrobial	1/9 (11.1%)		

**Table 3:** Relationship between age, Charlson comorbidity index, duration of antibiotic treatment, and mortality *Based on Mann-Whitney U tests SD, standard deviation

Variables	Deceased	Alive	p-value*
Mean age, in years ± SD	68.50 ± 2.12	53.96 ± 17.36	0.200
Mean Charlson comorbidity index, score ± SD	6.00 ± 2.83	1.83 ± 1.77	0.027
Mean duration of antibiotic treatment, in days ± SD	7.50 ± 9.19	11.59 ± 9.19	0.587

## Discussion

We performed a retrospective chart review of *S. lugdunensis* infections at King Abdulaziz University Hospital in Jeddah, Saudi Arabia, as no information was available in Saudi Arabia about the clinical disease, risk factors, antibiotic susceptibility, and outcomes for this particular organism. We identified 25 patients and found that the majority of positive cultures were from wound swabs, and most infections were community-acquired. Most patients had multiple comorbidities, and the Charlson comorbidity index was significantly higher in the deceased group than in the alive group.

In general, information on *S. lugdunensis* infections is limited [9,22-25], and in Saudi Arabia, only two cases of *S. lugdunensis* infections were ever reported [21,22]. The first case occurred in a pregnant woman where infection of the endometrium resulted in premature rupture of the membranes [22], and the second case started with a gluteal abscess that evolved into native triple valve endocarditis [21]. Both patients recovered after treatment.

Most of the patients in our study had community-acquired infections (68%). This finding is in contrast to a previous study that showed that community-acquired infection was the least common (10.4%) origin of infection [23] and a small study of 15 patients with clinically significant *S. lugdunensis* bacteremia, in which 33.3% of the patients had community-acquired infections [24].

Given that *S. lugdunensis* is a skin commensal, it is not surprising that 68% of our patients had skin and soft tissue infections. These infections also included surgical site infection, which makes sense as invasive procedures are known to contribute to such infections [23]. Most skin and soft tissue infections in our patients occurred below the lower abdomen, in the perineal area, and on the lower limbs, which points toward the areas that are heavily colonized with this organism. *S. lugdunensis* is known to colonize the perineal region preferentially [1] and has also been found in 140 plastic surgery patients to colonize the inguinal fold (unilateral in 22%, bilateral in 68%) [26].

An interesting finding was that the large majority (76%) of our patients were overweight or obese, which has not been reported before and may be a risk factor. The most common comorbidity that we came across was diabetes, followed by hypertension, heart disease, and renal impairment. A similar distribution of comorbidities was reported in a study from Taiwan, where hypertension was most common, followed by end-stage renal disease and diabetes mellitus [23].

In four of our bacteremia patients, the bacteremia was secondary to central line infection, and only one had primary bacteremia. This observation is similar to an observation in six patients with clinically significant bacteremia, five of whom had vascular catheters in the United States [27]. In contrast, in Taiwan, in 48 patients with clinically significant infections (41 of which had bacteremia), primary bacteremia (43.8%), was the most common source of infection [23]. This was different from what we found in our study, as there was a larger number that did not have an identifiable cause (primary bacteremia) of the bacteremia while most of our patients had a definitive source, either central line infections or other sources.

In terms of antibiotic susceptibility, we found that the majority of strains were susceptible to vancomycin (100%), the penicillin family member oxacillin (72%), clindamycin (64%), and trimethoprim-sulfamethoxazole (64%). In an extensive study of the antimicrobial susceptibility of *S. lugdunensis* strains (n=540) in Sweden, 99.6% of strains were susceptible to oxacillin (cefoxitin), 91.5% to clindamycin, and 99.8% to trimethoprim-sulfamethoxazole, while vancomycin was not tested [17]. In a smaller study in Taiwan, 90% of strains were susceptible to trimethoprim-sulfamethoxazole, 87% to clindamycin, and 80% to oxacillin [19], while a study from the US showed high resistance (45%) to penicillin [18]. Vancomycin resistance was not tested in the Swedish study, but vancomycin susceptibility was also found to be 100% in studies from Taiwan and the US [18,19]. These results show that large differences in susceptibility can exist between Saudi Arabia and other countries, so it is important to assess susceptibility when selecting treatment. Overall, most antibiotics worked on the isolates, and patients were treated successfully with piperacillin-tazobactam, amoxicillin-clavulanic acid, and other antibiotics. The mean duration of antibiotic treatment was 11.3 days, and this contributed to clinical improvement.

Two of our patients died within 30 days. One of these was a male with bacteremia and a Charlson comorbidity index of eight. The other was a female with a wound infection who received treatment with an inappropriate antimicrobial. Her cause of death was heart failure secondary to cardiomyopathy, and the immediate cause of death was pneumonia caused by *Streptococcus pneumoniae*. We cannot draw conclusions about mortality in general as most *S. lugdunensis* infection types do not result in the death of patients. Previous studies described high *S. lugdunensis* mortality when associated with infective endocarditis, which was initially reported to have a mortality of around 40-70% [1,10]. In more recent studies, the mortality due to infective endocarditis, regardless of the pathogen involved, was estimated to be 20% to 28% [28,29]. Only one recent study specifically reported *S. lugdunensis*-related infective endocarditis mortality, which was also low at 12.5% [30]. However, none of our patients had infective endocarditis. No mortality rates have been reported for *S. lugdunensis* infections in general.

Our study had several limitations. First and foremost, there were a limited number of patients due to the rarity of this infection, which did not allow us to perform a regression analysis for mortality. Another limitation is that we did not test for the presence of the *mecA* gene that confers resistance to, among others, oxacillin, as this test was unavailable in our hospital at the time of the study.

## Conclusions

*S. lugdunensis* caused clinically significant disease, especially in patients with multiple comorbidities, and a higher Charlson comorbidity index was found in deceased patients. Although *S. lugdunensis* infection accounts for a small number of patients, the clinical importance of the infection cannot be ignored and should be studied further to fully understand the burden of this disease and the clinical spectrum. We encourage further studies on a larger number of patients to fully understand the risk factors for the infection and the factors that contribute to the mortality rate.
